# The link between infant regulatory problems, temperament traits, maternal depressive symptoms and children’s psychopathological symptoms at age three: a longitudinal study in a German at-risk sample

**DOI:** 10.1186/s13034-017-0148-5

**Published:** 2017-03-06

**Authors:** Anna Sidor, Cristina Fischer, Manfred Cierpka

**Affiliations:** Institute for Psychosocial Prevention, University Clinic Heidelberg, Bergheimerstr. 54, 69115 Heidelberg, Germany

**Keywords:** Early regulatory problems, Psychopathological symptoms, Maternal depression, Families at risk

## Abstract

**Background:**

Difficult conditions during childhood can limit an individual’s development in many ways. Factors such as being raised in an at-risk family, child temperamental traits or maternal traits can potentially influence a child’s later behaviour. The present study investigated the extent of regulatory problems in 6-month-old infants and their link to temperamental traits and impact on externalizing and internalizing problems at 36 months. Moderating effects of maternal distress and maternal depressive symptoms were tested as well.

**Methods:**

In a quasi-experimental, longitudinal study, a sample of 185 mother-infant dyads at psychosocial risk was investigated at 6 months with SFS (infants’ regulatory problems) and at 3 years with CBCL (children’s behavioural problems), EAS (children’s temperament), ADS (maternal depressive symptoms) and PSI-SF (maternal stress).

**Results:**

A hierarchical regression analysis yielded a significant association between infants’ regulatory problems and both externalizing and internalizing behaviour problems at age 3 (accounting for 16% and 14% variance), with both externalizing and internalizing problems being linked to current maternal depressive symptoms (12 and 9% of the variance). Externalizing and internalizing problems were found to be related also to children’s temperamental difficulty (18 and 13% of variance) and their negative emotionality. With temperamental traits having been taken into account, only feeding problems at 6 months contributed near-significant to internalizing problems at 3 years.

**Conclusions:**

Our results underscore the crucial role of temperament in the path between early regulatory problems and subsequent behavioural difficulties. Children’s unfavourable temperamental predispositions such as negative emotionality and generally “difficult temperament” contributed substantially to both externalizing and internalizing behavioural problems in the high-risk sample. The decreased predictive power of regulatory problems following the inclusion of temperamental variables indicates a mediation effect of temperamental traits in the path between early regulatory problems and subsequent behavioural problems. Our results support the main effects of a child’s temperament, and to some degree maternal depressive symptoms, rather than the diathesis stress model of interaction between risky environment and temperamental traits.

*Trial registration* D10025651 (NZFH)

## Background

Difficult conditions during childhood can restrict an individual’s emotional, cognitive, and social development in multiple ways. There is evidence that children’s behavioural problems can be traced to infancy and early childhood, with the problems being more likely to ensue from rearing environments with a disposition of risk embedded in them [1]. According to the diathesis stress model, predispositional vulnerability in combination with stress makes individuals more susceptible to psychological disorders. In line with this model, exposure to high psychosocial risks, such as being raised in high-risk families (stress), and unfavourable temperamental traits (diathesis) are potential risk factors for behavioural problems later in life [ibid.].

### Infants’ regulatory problems

Early regulatory problems are construed as difficulties infants have in adjusting to the environment, regulating their behaviour and arousal and in self-soothing. These difficulties show up as symptoms typical for age and developmental stage of the child, such as crying, sleeping and feeding problems [2]. Crying in the first 3 months is regarded as the expression of the usual difficulty experienced in initial adjustment to childhood development [3]. However, according to the guidelines of the German Association for Child and Youth Psychiatry [4], *excessive crying* beyond the first 3–4 months of life is seen as a regulatory problem in early infancy. It influences the mother–child interaction and regulatory contexts such as self-soothing, sleeping and feeding. The prevalence rate of excessive crying in the first 3 months has been reported to range between 5 and 19% [5]. Persistence of crying beyond the third month has been reported only in 5.8% of the cases, and beyond the sixth month in 2.5% of them [6]. Around the third month, most children’s self-regulation abilities improve in a surge of development. During the course of early childhood, excessive crying can develop into other symptoms (e.g. sleep disorders) [7]. As with increased crying, temporary problems related to the *sleep*-*wake cycle* represent normal postnatal adjustment difficulties, such as the inability (generally accompanied by crying) to fall or stay asleep. With children being unable to fall asleep on their own, sleeping problems are attributed to insufficient parental support. The prevalence rate of early sleeping disorders in the first 2 years of life ranges between 10 and 30% [5, 8]. *Feeding problems* too are temporary disorders that occur during weaning and introduction of puréed and solid food to the diet. According to the guidelines of the German Association for Child and Youth Psychiatry, the signs of a feeding disorder are when feeding is perceived by the parents as stressful; a meal requires more than 45 min and/or the intervals between meals are less than 2 h [4]. The parent–child interaction during feeding is also strained. Due to fear of malnutrition, parents put pressure on the child, contributing to the perpetuation of feeding problems. Since meals in such cases require a great deal of time, the child is fed very frequently, and even during sleep, which results in a lack of appetite [5]. The prevalence rate of mild to moderate feeding disorders in the first 2 years of life is estimated to be 15–25% and serious disorders 3–10% [9].

### Temperament and self-regulation

According to Rothbart temperament has been defined as relatively consistent, constitutionally based individual differences in reactivity and self-regulation [10]. A biologically anchored basic facility, it develops due to aging processes and environmental influences in the interaction with caregivers [11]. Temperament is closely related to the excitation of the central nervous system and is seen as a biological foundation of later personality [12], influencing behaviour, the autonomous nervous system (sympathetic and parasympathetic nervous system functions) and activation of the cortex [11]. Rothbart’s definition of temperament can be measured in different ways. For this paper we used the approach of Buss and Plomin [13] which also includes a strong biological component, with it being phylogenetically rooted and determined to a great extent by hereditary. Their three constituent elements of temperament are emotionality, activity and sociability. Emotionality can be observed very early in infancy, with only negative aspects such as anxiety, fear, anger or sadness being recorded. The heritable biological anchor is the tendency towards being easily and intensely excited. The second element of temperament, activity, refers to behavioural arousal as motor activity, while sociability is perceived as a tendency, which overlaps with Eysenck’s notion of extraversion, to seek the company of other people [14]. Sociability has the highest (10-year) time stability, followed by activity, while emotionality appears to be less stable [13]. In summary, both theories support the assumption that temperament strongly determines the individual ability of emotional self-regulation. Infants’ regulatory disorders, such as excessive crying, sleeping or feeding problems, can be seen as indicators of “biologically rooted” difficult temperamental traits.

### Link between temperamental traits and regulatory difficulties

Previous research has linked excessive crying in infancy to temperamental traits such as negative emotionality or “difficult temperament” during toddlerhood. Stifter and Spinrad [15] show that excessively crying infants had higher levels of negative emotionality and a lower capacity for self-regulation at 5 and 10 months during a laboratory examination compared to “typical criers”. Wurmser and colleagues [7] reported that infants with a diagnosis of excessive crying at the age of 4 months were judged to be temperamentally more “difficult” at 30 months in comparison to other children. In the study of Wolke and colleagues [16], the negative influence was found until the primary school age (8–10 years), with parents judging the temperament of children who had cried excessively as babies higher on the “emotional-negative” and “difficult” scale. Similarly, Desantis and colleagues [17] found an association between duration of whining and unease in the first weeks of life, negative emotionality and externalizing disorders from 3 to 8 years of age. In another study the link between early regulatory problems and negative emotionality was mediated by maternal variables, such as maternal involvement and sensitivity [18].

It is important to note that there is an overlap between temperament and regulatory problems. Presumably, serious early regulatory problems are an expression of a “difficult temperament” with poor adjustment to the environment [7]. Ineffective regulatory mechanisms, stimulus hypersensitivity and deficits in behaviour regulation play a crucial role in both temperament and the development of regulatory disorders. Nevertheless, given the disparate roots of the two concepts, it is imperative to look at them separately. Temperament with a strong biological component is determined to a great extent by hereditary and regulatory disorders contain an additional interactional component between child and caregiver (learning experience).

### Influence of early regulatory problems on subsequent behavioural problems

Regulatory problems that persist longer than the first 3–4 months of life present a potentially unfavourable factor for further childhood development. The persistence and “broadening” of the child’s regulatory disorders into other areas of behaviour contribute to an increased risk of further social-emotional and cognitive impairment in infancy [15]. Large bodies of literature have sought to link early regulatory disorders to later behavioural problems. Wurmser and co-workers [7] report a greater frequency of both externalizing and internalizing problems (CBCL) among at 30 months old children who had cried excessively as babies. Scher and Zuckerman [19] found an association between frequent night waking in the first year of life and a higher CBCL score at 3½ years of age. However, the predictive validity of sleeping problems accounted for only 3% of the behaviour problem variance. In a study by Schmid and colleagues [20], persistent multiple regulatory disorders (increased crying, sleeping and feeding problems in the 5th month) predicted adjustment difficulties and a lack of social skills for pre-school children. This association applied, however, only to boys. The results of the Mannheim Child Risk Study [21] point to a more favourable overall prognosis for isolated regulatory disorders, with the rate of behavioural problems in later childhood being only slightly higher than that among children from the control group. Children with multiple regulatory disorders showed significantly higher rates of subsequent internalizing and externalizing disorders. These multiple regulatory disorders nevertheless played a minor role in comparison to the psychosocial pressures on the families included in the study. Children with the highest rate of mental problems had suffered not only multiple regulatory disorders as infants but had additionally a high psychosocial risks.

According to the meta-analysis of the link between infants’ regulatory problems and children’s later behavioural outcomes conducted by Hemmi and colleagues [22], persistent excessive crying has the greatest effect on subsequent symptoms such as externalizing problems, internalizing problems and ADHD, with feeding problems and multiple regulatory disorders being linked to general behavioural disorders. As observed in this study, infant sleeping problems had only a marginal influence on internalizing disorders, while the effect on ADHD was substantial.

### Link between temperament traits and child’s behavioural problems

The relationship between temperament and psychopathological symptoms in children is crucial for a better understanding of biological markers and regulatory processes involved in the emergence of psychopathological symptoms [23]. Child temperament is one of the important constitutional risk factors for behavioural problems, with a large body of evidence indicating the link between temperament in early childhood and behavioural problems in childhood and adolescence [24]. Childhood behaviour problems form two broad syndrome categories: *externalizing problems,* including undercontrolled behaviour, such as impulsivity, conduct problems, hyperactivity, and *internalizing problems* such as sadness, depression and anxiety [25]. Bates et al. [26] found that 7- to 8-year-old boys with *externalizing behavioural* problems had been rated as temperamentally “difficult” at 6 months of age. The lack of control at age 3 was the strongest predictor of externalizing behaviour at 9–15 years [27]. In a sample of 5- to 18-year-old boys with a CBCL Dysregulation Profile, e.g. high aggressive behaviour scores, Althoff and colleagues observed attention problems and anxious-depressive symptoms, a temperamental profile characterized by high novelty seeking, high harm avoidance, low persistence and low reward dependence [28]. As regards *internalizing problems*, many studies indicate their link to negative emotionality, characterized by high intensity and frequency of sadness, anger, discomfort and fear. Higher levels of negative emotionality in infancy and early childhood predict internalizing problems at 7 years of age [29]. High negative emotionality and low emotional self-regulation are risk factors for internalizing symptoms in preschool children (age 3–5 years). Negative affect has been seen as a predictor of anxiety when maternal personality characteristics interact to create a family environment with little emotional support for the child [30]. Gartstein, Putnam and Rothbarth found a link between high levels of negative emotionality and low levels of effortful control as well as both externalizing and internalizing problems [31].

In his review, Nigg [23] presents *different temperamental pathways* to specific forms of psychopathology, with, for instance, anxiety involving high negative emotionality and low effortful control, ADHD involving extremely low effortful control and conduct problems involving high anger. Lemery and colleagues found a link between temperament traits at 3.5–4.5 years and subsequent behavioural problems at 5.5 years. CBQ temperament scales such as anger, fear and sadness were positive predictors of both internalizing and externalizing problems, with anger as a better predictor of externalizing and Sadness of internalizing problems. Inhibitory control and attentional focusing were negative predictors of both domains of behavioural problems [32].

The data on the link between temperament traits and child’s behavioural problems involving *infants and very young children* are sparse. Examining low birth weight and premature infants for a 2-year period, Blair found negative temperament, assessed in the child’s first year of life, to be predictive of subsequent behavioural problems at the age of 3 years. Temperamental fear predicted later internalizing problems, whereas anger or frustration indicated subsequent externalizing symptoms [33]. In the study conducted by Northerner and colleagues negative emotionality at 1½ years predicted internalizing, externalizing and sleeping problems at 2 years [34]. Gartstein and colleagues found an association between high negative emotionality in infancy (3–9 months) and at 1½ to 3 years, and both externalizing and internalizing problems at kindergarten age (3–5 years) [31].

In the context of the construct overlap of *temperament and behavioural disturbances*, Niggs suggests that temperament and behavioural problems are not extensions of the same dimension despite the overlap [23]. Lemery and colleagues found measurements confounding in about 9% of temperament items and 23% of behavioural problem items, with the latter containing more temperament items than vice versa. Most importantly, the predictive power of temperamental traits remained high after the removal of confounding items from both domains, suggesting that the association between the two constructs is not only a methodological confounding issue [32].

### Environmental factors

In the transactional model, additional factors such as social environment are crucial for the emergence of psychopathological symptoms. According to the diathesis stress model [1], despite causing vulnerability to psychopathology, temperamental traits alone, without the co-occurrence of other environmental factors, may not be sufficient to trigger its full emergence. Social environment mediates the influence of temperament on the emergence of psychopathology: temperament may increase the likelihood of psychopathological disorder under high-risk conditions but has little effect in a low-risk environment [23]. Difficult temperament traits may lead to negative responses from caregivers and elicit conflict with peers. In a sample already exposed to putative risk factors, parents are likely to face increased problems coping with the challenges of children’s negative emotionality and temperamental difficultness. This “double strain” can lead to dysfunctional parenting practices, which in turn can increase the risk of behaviour problems. Laucht and colleagues [21] found the highest rate of mental problems among children who had suffered multiple regulatory disorders as infants and who were also exposed to high psychosocial risks. Children born in high-risk families appear to be generally more vulnerable to further stressors and maladaptive outcomes [35].

Parental psychopathology represents one of the potential risk factors for children’s behavioural problems. Children of depressed mothers tend to be more susceptible to psychopathology in childhood, adolescence, and adult life [36], being more socially withdrawn [37], less adept at developing age-appropriate social skills [38] and thus being less competent in forming peer relationships [39]. Young Mun et al. found temperamental traits, such as high reactivity, high activity and a short attention span at age 3–5 years, to be associated with externalizing problems at age 6–8 years, whereas withdrawal was found to be linked to internalizing problems, but only in children of parents with one of two lifetime psychopathology diagnoses [40]. Nelson and colleagues found the link between high levels of maternal depression and children’s behavioural problems at preschool age to end in the 1st grade [41]. Wurmser and colleagues observed a positive association between the CBCL scores for both externalizing and internalizing problems in former crying/fussing babies and their mothers with depressive symptoms at the children’s age of 30 months [7]. Lam, Hiscock and Wake [42] report higher maternal depression scores in 3- and 4-year-old children with externalizing and internalizing problems and current sleep disorders. These findings are in line with the meta-analysis of Goodman and colleagues [43], which shows an association between depression in mothers and children’s internalizing and externalizing problems, general psychopathology and negative emotionality. In poor and single-parent households, child age was found to be an important moderator, with effect sizes being stronger for younger children [ibid.].

### Study aims and hypothesis

The present study involves children who are raised in high-risk families and are more vulnerable to further stressors and maladaptive outcomes. The present study builds uniquely upon previous research by examining externalizing and internalizing problems in the context of regulatory disorders ant temperamental traits in a group of younger children raised in high-risk families up to the age of 36 months. The study investigates (1) the link between regulatory disorders and behavioural problems—the extent to which regulatory problems in 6-month-old infants have a negative influence on externalizing and internalizing problems at 36 months. The literature on this subject involving infants is limited, but given the findings of previous research, regulatory problems at 6 months are expected to be associated with a higher level of psychopathological symptoms at age 3. (2) The link between temperament and behavioural problems. We expect to find a positive association between behavioural problems and children’s temperamental traits such as negative emotionality and temperamental “difficulty” at the age of 3. (3) If early environment influences/moderates the link. According to the diathesis stress model [1], maternal depressive symptoms are expected to add to the link between children’s regulatory problems, temperamental traits and their psychopathological symptoms. The strength of this study lies in its attempt to assess the collective influence of early regulatory disorders and temperamental traits on children’s subsequent behavioural problems for a better understanding of psychopathological trajectories.

## Methods

### Participants

The sample comprised 184 at-risk mother–child dyads from the German family support research project “Nobody slips through the net” (KfdN) [44].^1^ One half of the families acted as an intervention group (IG, n = 92 at children’s age of 3 years) and took part in the early intervention program KfdN administered by midwives. The midwives visited the families on a regular basis for 1 year following birth, helping develop positive parent–child emotional relationships and co-regulative competences. The other half of the sample, the control group (CG, n = 92), though not supported in this particular way, received treatment as usual for families in Germany.

All the families were exposed to psychosocial risks owing to poverty (income below €1000 per household—IG 69.7%, CG 35%), lack of social/family support (IG 33.0%, CG 27.8%), excessive demands on the mother (IG 63.5%, CG 49.3%), mother’s mental health disorder (IG 36.9%, CG 31.3%), violence in the partnership (IG 16.9%, CG 5.2%), or underage mothers (IG 18.7%, CG 6.2%) (the data refer to the baseline T0).

### Study design

**Fig. 1:**
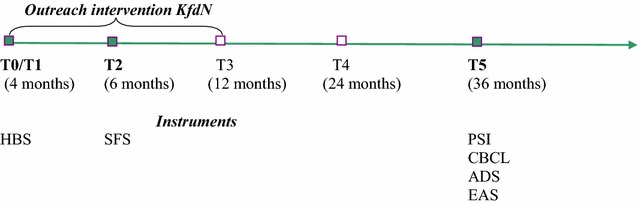
Study measurement points and instruments

The original research was conceived as a quasi-experimental, controlled longitudinal study under naturalistic conditions. The data used for the present study were collected at three intervals: the baseline (T0, N = 302), the second survey time point (T2, N = 289), when the children were on average 6.47 months old (SD = .65) (corrected due to prematurity), and at the fifth survey time point (T5, N = 184) at 36.70 months (SD = 1.14) (Fig. 1).

The dropout rate from the first to the fifth measurement points was 38.4% for the entire sample. The dropout group differed from the participants in several sociodemographic terms and was therefore selective. The mothers in the dropout group were on average significantly younger than those who continued to participate in the study (p < .001), they were also more likely to have no school-leaving qualification (23 vs. 14.6%), less likely to have graduated from a German Hauptschule (lower secondary education, ending at 9th grade) (54 vs. 34.5%), and graduated less often from a German Realschule (secondary education, ending at 10th grade) (19.7 vs. 27.2%) than their participating counterparts (p < .001). As regards net income, the mothers who still took part in the study at T5 had more money per month at their disposal compared to those who had dropped out of the study (p = .048).

The characteristics of the sample are described in Tables 1 and 2.

**Table 1:** Sociodemographic data on sample (mothers) at the baseline (child’s age 19 weeks)

	Intervention group	Comparison group	Significance
Age of mothers, M (SD)	24.5 (6.7)	28.2 (6.4)	p < .001

	n (n%)	n (n%)	
Marital status			
Married	29 (24.8%)	50 (39.1%)	p = .032
Single mother	24 (20.5%)	29 (22.7%)	
Single, partnership with the child’s father	61 (52.1%)	44 (34.4%)	
Single, a new partner	3 (2.6%)	5 (3.9%)	
Education			
Without qualification	27 (25%)	13 (10.4%)	p = .008
Secondary general school	47 (43.5%)	47 (37.6%)	
Intermediate secondary school	25 (23.1%)	39 (31.2%)	
Technical college entrance qualification	3 (2.8%)	5 (4%)	
University entrance diploma	4 (3.7%)	13 (10.4%)	
University	2 (1.9%)	8 (6.4%)	
Monthly income per household			
<€1000	76 (69.7%)	41 (35%)	p < .001
€1000 to €1500	9 (8.3%)	43 (36.8%)	
€1500 to €2000	15 (13.8%)	16 (13.7%)	
>€2000	9 (8.3%)	17 (14.5%)	
Nationality			
German	94 (83.9%)	99 (78%)	ns
Turkish	6 (5.4%)	7 (5.5%)	
Other	12 (10.8%)	21 (16.6%)	

*ns* not significant

**Table 2:** Children’s information at birth and at the baseline (child’s age 19 weeks)

	Intervention group, M (SD)	Comparison group, M (SD)	Significance
Born in which week of pregnancy (N^a^ = 292)	38.3 (2.80)	38.8 (2.27)	p = .06
Birth weight (g) (N = 300)	3031.82 (710.93)	3162.99 (615.39)	p = .09
Age T1 (corrected, in weeks) (N = 286)	19.3 (3.32)	19.0 (2.39)	ns

	n (n%)	n (n%)	
Gender (N = 300)	78 male (51.7%)	78 male (52%)	ns
Premature baby (birth < 37 SSW) (N = 292)	28 (19.2%)	16 (11.0%)	p = .05

^a^The variance of the *N* is based on different return ratios

*ns* not significant

### Measures

#### Child variables

The *infants’ regulatory problems* were recorded at T2 by means of a parent questionnaire on regulatory disorders in early infancy—“Questionnaire on crying, feeding and sleep (SFS)” [45]. The SFS refers to a “typical week” in everyday family life and can be applied within the first year of the child’s life. The Questionnaire contains 52 items (response mode: “1 never/seldom” to “4 always”): 3 to capture Wessel’s “rule of threes”, 24 for crying, whining and sleeping (e.g., cry duration, sleep latency), and 13 for feeding (feeding problems, concerns about the child’s weight), with the remaining 12 items assessing co-regulation, i.e. calming strategies that parents use when their child cries or when the child wakes up at night and cannot go back to sleep. The more difficulties children show in terms of crying, feeding and sleeping, the higher the SFS values. The assessment criteria of the questionnaire, which was a theoretical, factor-analytic model of analysis, were tested on a sample of 642 infants (both clinical and non-clinical subsamples). The factor analysis resulted in three easily interpreted areas: “crying, whining and sleep problems” (Cronbach’s α = .89), “feeding problems” (α = .82) and “co-regulation” (parental calming strategies against the child’s crying and sleep problems) (α = .81). With regard to validity, the SFS distinguished well between the clinical and non-clinical samples, with links being found to exist between the SFS and both diary entries and clinical diagnoses in the clinical sample (parent-infant consultation hours) [ibid.]. Because of our interest in regulation problems rather than strategies parents use when their baby cries, this study did not utilize the co-regulation scale.

Children’s *behavioural problems* were assessed at T5 with the German Version of the Child Behaviour Checklist for ages 1½ to 5 (CBCL 1½ to 5 [25, 46]). The CBCL assesses details of children’s “psychic functioning”, obtaining reports from parents, other close relatives, and/or guardians regarding children’s competencies and behavioural/emotional problems. The checklist consists of 100 items (response mode: “0 not true”, “1 somewhat or sometimes true” to “2 very true or often true”). The following seven syndrome scales are measured: “emotionally reactive” (Cronbach’s α = .73), “anxious/depressed” (α = .66), “somatic complaints” (α = .80), “withdrawn” (α = .75), “sleep problems” (α = .78), “attention problems” (α = .68), and “aggressive behaviour” (α = .92). In addition to the syndrome scales, CBCL1 ½ to 5 can be scored on two groups of syndromes, “internalizing” (α = .89) and “externalizing” (α = .92) and the global scale “total problems” (α = .95). Subsequent test-retest-reliability scores (8-Day) were obtained for “internalizing” (r = .90), “externalizing” (r = .87), and “total problems” (r = .90). In terms of discriminant validity, the CBCL correctly classified 84.2% of the children, 7.3% of whom were overreffered (i.e. false positive) and 8.6% were underreffered (false negative).

Children’s *temperament* was assessed by means of the emotionality-activity-sociability-temperament survey EAS [13, 47], with the questionnaire measuring temperamental characteristics such as “emotionality”, “activity”, “sociability” and “shyness”. The EAS is a reliable instrument for evaluating temperamental traits with satisfactory to good internal consistency values (Cronbach’s α: Emotionality α = .72, Activity α = .72, Shyness α = .83) except for Sociability (α = .59) and a good interrater correspondence (Spearman–Brown corrected intraclass correlations for emotionality .57, for activity .60, for shyness .68 and for sociability .56). The data refer to the measurement time T5.

#### Environmental variables

The families’ *general exposure to risk* was measured with the help of the “Heidelberger Belastungsskala” (HBS, Heidelberg Stress Scale) [48]. The HBS measures a family’s stress in the following areas: child stress, parent/family stress, social burden and financial burden, with the values ranging between 0 (no stress) and 100 (very high stress). The following range allocations were set using the HBS: range 0–20: no stress; 21–40 small to moderate stress; 41–60: middle stress; 61–80 high stress; 81–100 extremely high stress. The HBS shows an excellent interrater reliability within a homogeneous professional group (psychology students) (ICC = .92). As regards construct validity, significant correlations were found with both maternal sensitivity (CARE-Index) (r = −.20; p = .001) and maternal distress (PSI) (r = .14, p = .05), while, in case of predictive validity, the risk of taking the child into care in case of high stress in the HBS was increased by 4.5 times (ibid.). The data refer to the T0 measurement time.

The Allgemeine Depressionsskala (ADS, General Depression Scale) [49] was used to measure *maternal depressive symptoms* at T5. This is a 20-item screening instrument with a 4-level answer format (“seldom”, “sometimes”, “often” and “most of the time”). The cut-off value of the instrument for a clinically relevant depressive disorder is 23. The internal consistency with α = .89, the high concordance with beck depression inventory (BDI) and hamilton depression scale (HAM-D) and the fair discriminant validity of the instrument are considered definite.

The short form of the German version of the standardized parental questionnaire PSI–SF (“parental stress index short form”, [50]) was used to measure *maternal stress*. This short form consists of 36 items, for which the answer format ranges on a five-level scale from “strongly agree” to “don’t agree at all.” The questionnaire is divided into three subscales: the “parental distress” scale (α = .87), the “dysfunctional parent–child interaction” scale (α = .80), and the “difficult child” scale (α = .85).

### Participant recruitment and procedure

Given the objectives of the study, the participants were required to meet the following selection criteria: Members of both the intervention and comparison groups were required to be in stressful circumstances owing to psychosocial risk factors (see “Participants” section), which needed to be at least “moderate” (HBS > 20, see Measurement Instruments). Families in the intervention group had to live in the program area (Saarland, administrative districts Bergstrasse and Offenbach in Hesse, or the city of Heidelberg) and be supported by a KfdN family midwife, while the burdened families in the comparison group could not be from the KfdN intervention areas named above, since families at risk were intended to be reached as extensively as possible in the KfdN areas. Furthermore, the comparison group families could not have been involved in an intervention that could be compared with the intervention by the family midwives in the project area.

Following recruitment of the comparison group, we approached institutions such as maternity clinics, welfare offices, pregnancy counselling services, midwife practices, paediatricians, family support institutions, counselling centres, etc., in other districts of Baden-Württemberg, Rheinland-Pfalz, and Hesse, which were likely to have contact with burdened pregnant women and mothers with newborn children. If we agreed upon a potential family, we sent the relevant contact details to the staff members of the study. Families in the KfdN group were recruited through midwives. Upon agreement regarding participation in the study, the contact details of families from both groups were forwarded to the staff members. As soon as the informed consent was signed by a family, a specially trained student assistant contacted them. The participating mothers were informed about the study and data protection regulations during the first appointment in their own homes, with the families having to formally agree to the data protection terms and conditions. Following this, the stress level was assessed (HBS, T0). At the child’s age of about 6 months (T2), the assistants contacted the participating families to make an appointment for the second measurement point, at which SFS was to be filled out. Around the child’s third birthday, our assistants once again telephoned the participating families to agree upon an appointment for the fifth measurement point (T5). Parents completed a set of surveys including the CBCL, the ADS, the EAS and the PSI.

The varying numbers of test participants within the presented variables are the result of varying response rates.

#### Statistical analyses

For the multivariate prediction of externalizing and internalizing behavioural problems at T5 (CBCL), regulatory problems at T2 (SFS) and child’s temperamental traits (EAS) at T5 were entered step by step into a hierarchical regression equation (method enter) intended to determine their unique contributions to the variance explanation (R^2^ change). Potential confounding/control variables such as maternal education level, household income, global risk score, infant’s gender and group affiliation (IG vs. CG) were included in the model and fitted in the equation. Maternal distress and her depressive symptoms at T5 as variables were also taken into account. Potential moderator effects of the depressive symptoms in interaction with children’s temperamental traits were included in the last step (interactions “maternal depression X difficult child” and “maternal depression X emotionality”).

The potential differences between the two groups (IG and CG) in terms of continuous variables were tested by means of the Mann–Whitney U Test owing to the unfulfillment of the normal distribution requirement (Kolmogorov–Smirnov-test significant, see Table 3).

**Table 3:** Descriptive statistics on SFS scales (T2, child’s age 6 months), CBCL 1½ to 5 scales (T5, child’s age 3 years), EAS scales (T5), ADS (T5) and PSI-Scales (T5)

	Intervention group, M (SD)	Comparison group, M (SD)	Comparison between groups (U test)	Normal distribution (whole group) (K-S-Z)
SFS crying, whining and sleep problems T2	1.54 (.31)	1.56 (.30)	ns	***
	n = 143	n = 150		
SFS feeding problems T2	1.22 (.29)	1.23 (.29)	ns	***
	n = 143	n = 150		
CBCL internalizing problems T5	9.40 (6.30)	7.48 (6.11)	*	+
	n = 77	n = 83		
CBCL externalizing problems T5	12.66 (8.25)	11.56 (7.58)	ns	ns
	n = 82	n = 87		
EAS emotionality T5	2.63 (.85)	2.64 (.82)	ns	***
	n = 89	n = 92		
EAS activity T5	4.14 (.64)	4.05 (.64)	ns	***
	n = 89	n = 92		
EAS sociability T5	3.85 (.58)	3.81 (.56)	ns	***
	n = 89	n = 92		
EAS shyness T5	2.20 (.70)	2.24 (.70)	ns	***
	n = 88	n = 92		
ADS (mothers) T5	13.84 (9.69)	13.68 (10.17)	ns	***
	n = 89	n = 91		
PSI parental distress T5	2.17 (.77)	2.26 (.86)	ns	***
	n = 92	n = 92		
PSI dysfunctional parent–child interaction T5	1.58 (.50)	1.54 (.46)	ns	***
	n = 92	n = 92		
PSI difficult child T5	2.05 (.71)	2.09 (.65)	ns	***
	n = 91	n = 92		

*SFS* questionnaires on crying, feeding and sleeping, *CBCL* child behavior checklist, *EAS* emotionality-activity-sociability-temperament survey, *ADS* Allgemeine Depressionsskala, *PSI* parental stress index, *K-S-Z* Kolmogorov–Smirnov test, *U test* Mann–Whitney-U test, *ns* not significant

* p ≤ .05

** p ≤ .01

*** p ≤ .001

^+^p ≤ .10

Additionally, Pearson’s correlations were computed for an overview of associations between continuous parameters (SFS, CBCL, EAS) as well as for testing potential multicollinearity among independent variables. For all calculations, a significance level of .05 was determined (two-tailed). The statistical analysis of the data was conducted using the statistics program SPSS for Windows, Version 21.0.

## Results

### Descriptive statistics

Table 3 shows descriptive statistics for all variables applied. As no differences between the two subgroups, intervention and comparison, were found, they were combined for all subsequent analyses.

### Correlations between SFS at T2 and CBCL 1.5–5, EAS, PSI and ADS at T5

Table 4 shows significant correlations between the following tested parameters at T5: child’s internalizing and externalizing problems correlated positively with child’s temperamental traits negative emotionality and shyness and negatively with child’s sociability. Only internalizing problems were correlated negatively with child’s activity. Maternal depressive symptoms were positively associated with child’s negative emotionality and negatively with activity and sociability. Maternal depressive symptoms correlated positively with child’s internalizing and externalizing problems.

**Table 4:** Bivariate correlation coefficients (according to Pearson) for infant’s regulation problems (6 months, T2), children’s temperamental treats EAS (3 years, T5) and their psychopathological internalizing/externalizing symptoms CBCL 1½ to 5 (T5)

	SFS C/S T2	SFS F T2	EAS emo T5	EAS act T5	EAS soc T5	EAS shy T5	CBCL intern T5	CBCL extern T5	ADS T5	PSI PD T5	PSI DI T5	PSI DC T5
SFS C/S T2	1											
	N = 248											
SFS F T2	.39***	1										
	N = 274	N = 248										
EAS emo T5	.27***	.23**	1									
	N = 175	N = 175	N = 181									
EAS act T5	−.18*	−.17*	−.25**	1								
	N = 175	N = 175	N = 181	N = 181								
EAS soc T5	−.16*	ns	−.25***	.32***	1							
	N = 175		N = 181	N = 181								
EAS shy T5	ns	ns	.162*	−.338***	−.498***	1						
			N = 180	N = 180	N = 180	N = 180						
CBCL intern T5	.26***	.25***	.51***	−.19*	−.31***	.30***	1					
	N = 156	N = 156	N = 154	N = 154	N = 154	N = 153	N = 160					
CBCL extern T5	.32***	.28***	.55***	ns	−.25**	.20*	.72***	1				
	N = 164	N = 164	N = 162		N = 162	N = 161	N = 153	N = 169				
ADS T5	.37***	.22***	.43***	−.19*	−.29***	ns	.36***	.41***	1			
	N = 174	N = 174	N = 178	N = 178	N = 178		N = 154	N = 161				
PSI PD T5	.36***	.29***	.49***	−.26***	−.30***	.19*	.39***	.42***	.71***	1		
	N = 178	N = 178	N = 178	N = 181	N = 181	N = 180	N = 156	N = 165	N = 180			
PSI DI T5	.33***	.22***	.39***	−.16*	−.20***	.21**	.59***	.58***	.45***	.57***	*1*	
	N = 178	N = 178	*N* *=* *181*	N = 181	N = 181	N = 180	N = 156	N = 165	N = 180	N = 184		
PSI DC T5	.39***	.31***	.68***	−.21**	−.23***	.16*	.57***	.66***	.55***	.66***	.69***	1
	N = 177	N = 177	*N* *=* *181*	N = 181	N = 181	N = 180	N = 156	N = 164	N = 180	N = 183	N = 183	

SFS, *C/S* crying/sleep, *F* feeding, *EAS emo* emotionality, *act* activity, *soc* sociability, *shy* shyness, *CBCL intern* internalizing, *extern* externalizing, *PSI PD* parental distress, *DI* dysfunctional parent–child interaction, *DC* difficult child, *ns* not significant

* p ≤ .05

**  p ≤ .01

*** p ≤ .001

Maternal distress correlated positively with the child’s negative emotionality and shyness and negatively with both activity and sociability. Maternal distress correlated positively with both children’s internalizing and externalizing problems.

Dysfunctional mother–child interaction correlated positively with the child’s negative emotionality and shyness and negatively with both activity and sociability. Dysfunctional mother–child interaction correlated positively with child’s internalizing and externalizing problems.

Child’s temperamental difficulty correlated positively with child’s negative emotionality and shyness and negatively with both activity and sociability. Child’s temperamental difficulty correlated positively with both internalizing and externalizing problems.

As regards regulatory problems at T2, crying and sleeping problems in the infancy were positively associated with the concurrent feeding problems. Crying and sleeping problems were positively associated with child’s internalizing and externalizing problems and with child’s negative emotionality at the age of 3. Crying and sleeping problems were negatively associated with child’s activity and sociability at the age of 3. Feeding problems in the infancy were positively associated with both internalizing and externalizing problems at the age of 3. Feeding problems were positively associated with child’s negative emotionality and negatively associated with child’s activity.

### Prediction of internalizing problems (CBCL) at 3 years (T5) by means of regulatory problems at 6 months (T2), maternal distress, maternal depressive symptoms and child’s temperament traits

Crying/sleep and feeding problems at T2 were significant predictors of internalizing problems at 3 years (Beta = .20, p ≤ .05 and Beta = .26, p ≤ .01 respectively), contributing to 14% of the variance. Maternal depressive symptoms at T5 significantly improved the explanation for children’s internal symptoms, contributing to 9% of variance (Beta = .34, p < .001). The PSI scales strongly improved the explanation contributing to 13% of variance of internal problems: “Difficult child” was a significant predictor (Beta = .47, p < .001). The child’s temperamental traits had a small yet significant contribution of 5% of the variance. Negative emotionality was a significant predictor of internalizing problems (Beta = .22, p ≤ .05), with the temperamental shyness having a near-significant contribution (Beta = .16, p < .10). Other temperamental traits, children’s gender, the sociodemographic variables and global risk were not significant. Neither interaction terms—“maternal depression X difficult child” and “maternal depression X emotionality”—contributed to the variance of internalizing behaviours.

In the final model, only the temperamental traits “Difficult child” (Beta = .32, p < .05) and negative emotionality (Beta = .22, p ≤ .05), together with near-significant contributions of shyness (Beta = .16, p < .10) and feeding problems (Beta = .16, p < .10), added to internalizing problems at 3 years, whereas crying and sleeping problems and maternal depressive symptoms were not significant. This suggests that children’s temperamental traits explained their internalizing problems better than early regulatory difficulties. The final model explained 39% of the variance in the children’s internalizing problems at the fifth measurement point (R^2^ = .46; corrected R^2^ = .39; F = 6.22; p < .001) (see Table 5).

**Table 5:** Linear regression analysis for investigating effects of infant regulatory problems at 6 month, maternal depression and distress and child’s temperament traits on its internalizing problems at 3 years (N = 123)

Model summary	R^2^ change	R^2^	Corrected R^2^	F	Beta
*Block 1/SES, global risk*	ns	.028	.003	ns	
Mother’s education					ns
Household income					ns
Global risk score (HBS)					ns
*Block 2*	ns	.036	.003	ns	
Child’s gender					ns
*Block 3*	ns	.053	.013	ns	
Group (IG/CG)					ns
*Block 4/early regulatory problems SFS*	.*143*	.196	.148	4.046***	
Crying/sleeping problems at 6 months					.202*
Feeding problems at 6 months					.257**
*Block 5/maternal depression ADS*	.*090*	.286	.237	5.766***	
ADS at 3 years					.337***
*Block 6/PSI scales*	.*130*	.416	.359	7.264***	
Parental distress at 3 years					ns
Dysfunctional parent–child-interaction at 3 years					ns
Difficult child at 3 years					.467***
*Block 7/child’s temperament EAS*	.*047*	.464	.389	6.223***	
Emotionality at 3 years					.221*
Activity at 3 years					ns
Sociability at 3 years					ns
Shyness at 3 years					.*164* ^+^
*Block 8/interaction maternal depression X child’s temperament*	.003	.467	.381	5.453***	
ADS X Difficult child					ns
ADS X Emotionality					ns

Significance of change in F for each signficant values are indicated in italics

*SFS* questionnaires on crying, feeding and sleeping, *CBCL* child behavior checklist, *EAS* emotionality-activity-sociability-temperament survey, *ADS* Allgemeine Depressionsskala, *PSI* parental stress index, *ns* not significant

*** p ≤ .001; ** p ≤ .01; * p ≤ .05; ^+^ p ≤ .10

### Prediction of externalizing problems (CBCL) at 3 years by means of regulatory problems at 6 months, maternal distress, maternal depressive symptoms and child’s temperament traits

Both crying/sleep and feeding problems at T2 were significant predictors of internalizing problems at 3 years (Beta = .28, p < .001 and Beta = .19, p ≤ .05 respectively), contributing to 16% of the variance. The addition of maternal depressive symptoms to the model helped improve the explanatory power, contributing to 12% of the variance (Beta = .38, p < .001). Inclusion of the PSI scales improved the model’s explanatory power independently and significantly, contributing to 18% of the variance: “Difficult child” was a significant predictor of children’s externalizing problems (Beta = .56, p < .001). The children’s temperamental traits improved the model’s explanatory power and contributed to 6% of the variance, with negative emotionality and activity proving to be positive predictors (Beta = .22, p < .05 and Beta = .20, p < .01 respectively), whereas shyness contributed only near-significant (Beta = .13, p < .10). Children’s gender accounted for a separate small contribution of 4% of the variance (Beta = −.20, p < .05). The global risk score made a separate near-significant contribution (Beta = .17, p < .10). Children’s sociability and maternal demographic variables didn’t add any explanatory power. Neither interaction terms—“maternal depression X difficult child” and “maternal depression X emotionality”—contributed to the variance of externalizing difficulties.

In the final model, only temperamental traits “Difficult child” (Beta = .41, p < .01), negative emotionality (Beta = .22, p < .05), activity (Beta = .20, p < .01) and shyness (Beta = .13, p < .10) contributed to externalizing problems at 3 years, whereas feeding, crying and sleeping problems and maternal depressive symptoms were not significant. Again, negative temperamental traits explained children’s behavioural problems better than their regulatory difficulties in infancy. The final model explained 56% of the variance in children’s externalizing problems at 3 years (R^2^ = .61; corrected R^2^ = .56; F = 11.75; p < .001) (see Table 6).

**Table 6:** Linear regression analysis for investigating effects of infant regulatory problems at 6 months, maternal depression and distress and child’s temperament traits on its externalizing problems at 3 years (N = 128)

Model summary	R^2^ change	R^2^	Corrected R^2^	F	Beta
*Block 1*/*SES, global risk*	.*066*	.066	.043	2.92*	
Mother’s education					ns
Household income					ns
Global risk score (HBS)					*.165* ^+^
*Block 2*	.*037*	.103	.074	3.55**	
Child’s gender					−.196*
*Block 3*	.000	.103	.066	2.82*	
Group (IG/CG)					ns
*Block 4 early regulatory problems SFS*	.*158*	.261	.218	6.098***	
Crying/sleep problems at 6 months					.287***
Feeding problems at 6 months					.190*
*Block 5*/*maternal depression ADS*	.*117*	.378	.336	9.113***	
ADS at 3 years					.384***
*Block 6/PSI scales*	.*176*	.554	.512	13.232***	
Parental distress at 3 years					ns
Dysfunctional parent–child-interaction at 3 years					ns
Difficult child at 3 years					.562***
*Block 7*/*child’s temperament*					
*EAS*	.*055*	.609	.558	11.754***	
Emotionality at 3 years					.220*
Activity at 3 years					.199**
Sociability at 3 years					ns
Shyness at 3 years					*.130* ^+^
*Block 8/maternal depression X child’s temperament*	.001	.610	.551	10.226***	
ADS X difficult child					ns
ADS X emotionality					ns

Significance of change in F for each signficant values are indicated in italics

*SFS* questionnaires on crying, feeding and sleeping, *CBCL* child behavior checklist, *EAS* emotionality-activity-sociability-temperament survey, *ADS* Allgemeine Depressionsskala, *PSI* parental stress index, *ns* not significant

*** p ≤ .001; ** p ≤ .01; * p ≤ .05; ^+^ p ≤ .10

## Discussion

The aim of this study was to examine the extent to which regulation problems in infants at 6 months account for their behavioural problems at 36 months, taking into account children’s temperament traits and environmental factors such as maternal depression/stress and economic disadvantage.

### The link between regulatory problems in infancy and externalizing and internalizing problems at age three

In line with other research [7, 15–22], our findings indicate an association between early regulation difficulties and children’s behavioural problems. Controlling for the net income per household, mother’s educational level and child’s gender, we have observed a significant association between crying, whining and sleeping problems at 6 months and both externalizing and internalizing problems at age three. In the present study, 16% of the variance in children’s externalizing problems and 14% in internalizing problems were explained by infant regulatory difficulties during the 6th month. However, after adding temperamental traits to the model, only feeding problems remained as a near-significant predictor of internalizing difficulties. No link between early regulatory problems and externalizing problems was found. This is in line with previous research, which has also shown insufficient negative influence of early regulatory disorders. For instance, the predictive validity of sleeping problems in the first year has accounted for only 3% of the variance in behaviour problems (CBCL) at 3.5 years [19]. In a cohort study, persistent sleeping disorders in the first year accounted for only 1.4% of the variance of CBCL at 2 years [51]. In their meta-analysis, Hemmi and colleagues [22] report small to medium effects on both internalizing and externalizing problems.

One of the main reasons why temperament traits were stronger predictors of behavioural problems than regulatory problems might be that regulatory problems were measured earlier than temperament and behavioural problems. The cross-sectional measurement of the last two constructs shows a strong association between them. The concurrent measure of “difficult” temperament is likely more qualified to explain children’s behavioural problems than regulatory difficulties in infancy.

The etiological mechanisms involved in early regulatory problems’ long-term effects on subsequent emotional or behavioural difficulties in children remain unclear. Presumably, serious early regulatory problems are an expression of a “difficult temperament” with poor adjustment to the environment [7]. Excessive crying beyond the 3rd month is regarded as an indicator of dysfunctional regulatory capacities and likely low behavioural inhibition, predictive of subsequent behavioural problems [16]. Ineffective regulatory mechanisms, stimulus hypersensitivity and deficits in behaviour regulation purportedly play an important role in both “difficult temperament” and the development of regulatory disorders (see overview [22]). Temperamentally rooted low levels of regulative factors such as behavioural control and inhibition make children susceptible to both early regulatory problems in the infancy and psychopathological outcomes in the middle childhood. On the other hand the decreased influence of early regulatory difficulties is due, presumably, to the common variance of regulatory problems and temperament, with both domains possessing self-regulatory capacities. Our observation regarding a mediation path through temperament traits supports this hypothesis (see the next section).

Only feeding problems remained as a near significant predictor of internalizing difficulties, most likely due to different mechanisms involved and to a smaller overlap with the temperamental factors in comparison to crying/sleeping problems. Ineffective regulatory mechanisms probably play in feeding problems only a subordinate role in comparison to factors such as strained parent–child feeding-interaction or the lack of appetite regulation.

### The link between children’s temperamental traits and their behavioural problems

Our results underscore the crucial role of temperament in the path between early regulatory problems and subsequent behavioural difficulties. In conformity with other findings [i.e. 10, 26–34], temperamental traits contributed substantially to the child subsequent behavioural problems at the age of 36 months. Maternal assessment of the child as “difficult” explained 18% of the variance of externalizing and 13% of internalizing problems. The unique contribution of the EAS scales was smaller, likely due to multicollinearity with the PSI scale “Difficult child”, accounting for respectively 6% of externalizing and 5% of internalizing problems. According to Abidin [50], this scale captures disorders that are caused by temperament or are rooted in self-regulation difficulties.

The EAS scale “Emotionality” was associated with both externalizing and internalizing problems. Similarly, Gartstein, Putnam and Rothbart [31] found negative emotionality to be linked to behavioural problems in young children. In the study of Northerner and colleagues involving toddlers, negative emotionality had a particularly salient influence on children’s early behavioural problems, even when accounting for their families’ levels of risk and other temperament traits [34]. This temperamental trait, characterized by a general instability, high reactivity, fear, frustration, anger and sadness, has been linked to the personality trait of neuroticism in adulthood [31]. “Difficult temperament” is characterized by intense reactivity, lability, negative mood expression such as outbursts of crying or aggression and slow adaptability [53], containing both, negative emotionality and low levels of self-regulation, such as effortful control [24].

These temperamental predispositions constitute the aetiology of children’s psychopathology; the involved mechanisms, however, remain unclear. High levels of reactivity and negative emotionality connected to low levels of regulative temperament factors such as effortful control make children susceptible to psychopathological outcomes [24]. Highly emotional, fearful children are more prone to anxiety disorders, while those who are habitually sad are susceptible to depressive symptoms, and children characterized by anger/frustration run a greater risk of developing a disruptive behaviour disorder [ibid.].

Our observations shed new light on the link between early regulatory problems and behavioural difficulties. The decreased predictive power of regulatory problems, with temperamental variables having been factored in, points to a partial mediation effect of temperamental traits in the path between early regulatory problems and subsequent behavioural difficulties. The concurrent measure of “difficult” temperament is likely more qualified to explain children’s behavioural problems than regulatory difficulties in infancy. Another aspect is a strong construct overlap of temperament and behavioural disturbances. Nevertheless, results demonstrate that behavioural problems are not just an extension of difficult temperament [23, 32]. The methodological issues cross -sectional measurement and the same measurement method (maternal report) should be taken into account as well.

In line with other findings, in this study, high levels of activity were associated exclusively with externalizing problems [i.e. 40]. Berdan et al. [54] found that highly active preschool children are at risk of exhibiting behaviour problems in kindergarten. Immoderate levels of activity are seen as one of the markers of extraversion, and children with elevated levels of extraversion can be characterised as highly active and constantly exploring their environment. Children who are very active can exhibit these behaviours in a maladaptive manner, showing frustration and aggression when their goals are blocked. Young children high on surgency/extraversion have been seen to use aggressive strategies to overcome barriers when seeking something perceived as highly rewarding [55].

### Testing the role of early environment on child’s behavioural problems

Consistent with other findings [36–43], both externalizing and internalizing problems at the age of 36 months were found to be associated with concurrent maternal depressive symptoms, contributing respectively to 12 and 9% of the variance of the children’s behavioural problems. The impact of elevated maternal depression scores on young children’s psychopathological symptoms could be interpreted along the lines of the meta-analysis of Goodman and colleagues [43], who found the strongest effect sizes of parental psychopathology for families with lower income and younger children. Similarly, Nelson et al. found a link between high levels of maternal depression and children’s behavioural problems at preschool age [41]. With the addition of temperament variables, however, the predictive power of maternal depressive symptoms disappeared. This may be seen as a mediation effect of temperamental traits underscoring their crucial role in the development of behavioural problems.

Maternal depressive symptoms have been found to be linked to infants’ “difficult” temperamental traits, with difficult infant’s temperament being a predictor of maternal depression [56, 57]. In our study also, the bivariate relationship between maternal depressive symptoms and child’s temperamental difficulty at age three was found to be pronounced, although the direction of the association was uncertain. It is important to keep in mind that mothers’ perception of their infants’ behaviour happens to play a role in the association between maternal depression and child temperament. The maternal perception of child’s conduct is strongly influenced by the mother’s general frame of mind. It can be assumed that mothers who score higher on a depression scale are likely to overestimate their children’s “difficult” behaviour due to a negative cognitive bias.

Demonstrating the link between maternal depressive symptoms and both externalizing and internalizing behaviours, our findings contribute to the multifinality model in developmental psychopathology [58]. The results of our study show a stronger impact of maternal depression on externalizing problems compared to the internalizing ones, which is likely indicative of the difficulties children of depressed mothers have with emotional regulation of aggression [43]. One explanation for the link between maternal depression and children’s internalizing problems might be that children of depressed mothers may have higher levels of negative emotionality and lower levels of positive emotionality, both of which, together with genetic and social learning pathways, may predispose them to developing depression [ibid.].

Although parental psychopathology has been discussed as a moderator between child temperament and behavioural problems [40], we did not observe any moderating effects of maternal depression in the interaction with negative temperamental traits. Depressed mothers likely find the task of parenting to be overwhelming, especially when children are temperamentally “difficult”. Children’s difficult temperament alongside behavioural problems affect maternal well-being, eliciting negative rearing behaviour such as inconsistency or some other restrictiveness, which in turn can aggravate children’s behavioural problems [24]. On the other hand, mothers who score higher on a depression scale are likely to overestimate their children’s negative behaviour. Our results, however, tend to support the main effects of child temperament, and to some extent maternal depressive symptoms, rather than the diathesis stress model of interaction between risky environment (maternal depression) and temperamental traits.

Current maternal distress did not contribute to the variance explanation. In bivariate analyses, however, both externalizing and internalizing problems were found to correlate with the PSI scale “Parental distress”. In the regression model, maternal depressive symptoms contributed strongly to the explanation of the variance of children’s externalizing and internalizing problems, whereas the role of parental distress was not significant, which was likely due to multicollinearity with the depressive symptoms. A strong correlation between maternal depressive symptoms and maternal distress suggests that both self-report methods, ADS and the PSI scale “parental distress”, possibly measure quite similar constructs, with mothers’ depressive symptoms being strongly related to their distress.

Maternal assessment of interaction with the child as being dysfunctional was not a significant predictor. Again, the bivariate association with behavioural problems was pronounced, but couldn’t be found in the regression model due to multicollinearity with other variables. It is known, however, that the quality of child-parent relationship moderates the influence of biological adversities such as prematurity or adverse temperamental dispositions on children’s outcomes. Supportive parenting can buffer those adversities, whereas a less supportive environment exacerbates biological risks [52].

The family global risk exposure had only a near-significant effect on children’s externalizing behaviour. This rather small effect can be explained by the low variability in our sample, characterised by low socioeconomic background and high psychosocial risks.

### Limits of the study

The direction in which children’s temperamental traits are shown to influence their behaviour problems in a regression model may be questionable as this data were gathered at the same measurement point. The regression model was used to test both the influence of early regulatory problems (longitudinal) and temperament traits on behavioural problems at kindergarten age, with the results, in terms of the impact of regulatory problems on temperament, being interpreted only as an association as opposed to a prediction. Unfortunately, we did not gather data on infants’ temperament, and thus it was not possible to assess the impact of early temperament traits and regulatory problems on subsequent behavioural difficulties. The direction of influence of maternal depressive symptoms as predictors of the CBCL scales in the regression model may also prove contentious and may be interpreted in a bidirectional manner. A poor internal consistency of the EAS scale Sociability (α = .59) represents a further methodological limit. However, this scale did not play a significant role in our hypothesis.

The issue of multicollinearity is prevalent, as we used many predictors in the same regression model, some of them strongly overlapping. We calculated a correlation matrix and used step- by- step hierarchical regression for the better control and understanding of the multicollinearity issue.

Given that our study deals with a low SES-at-risk sample, the generalizability of its results is limited. Besides our selective sampling and the corresponding lack of a normative control sample, it can also be assumed that the study subjects, who were exposed to psychosocial stress, had difficulties while filling out the questionnaires, which could have contributed to distortions in the response behaviour.

Two additional and important aspects of self-regulation are effortful control and quality of parenting, neither of which has yet been tested in our sample. However, the next measuring point at elementary school age has been planned, when, among other things, data on children’s effortful control and parental rearing behaviour will be gathered.

### Clinical implications

Our findings provide evidence of a negative influence of difficult temperamental traits and early regulation problems on children’s psychic health. It is imperative, therefore, that there is a concerted effort (on the part of healthcare professionals in particular) toward enhancing the general awareness of the sensitive period young mothers experience and providing relevant support to them. Mothers in an at-risk population are likely to be more challenged by difficulties with their children and have fewer resources, such as social support or access to counselling services, in comparison to their more fortunate counterparts. This in turn may contribute to the broadening of the children’s initial regulatory problems. In case of severe regulatory difficulties, it is advisable to draw parents’ attention to the parent-infant advisory services, which can not only help improve early childhood regulatory problems but also facilitate mother–child interaction and help relieve pressure on young families. An easy access to support services provided by e.g. family health visitors, particularly in the so-called “high risk families”, is recommended. Services offering early assistance following childbirth (e.g. the KfdN prevention project [44] or comparable projects) have proved to be effective in improving children’s social development as well as reducing dysfunctionality in mother–child interaction [59] and, thus, can be a valuable addition to the outreach initiatives.

## Conclusion

In summary, our results demonstrate that children’s temperamental predispositions, paired with a history of regulatory problems in infancy and maternal depressive symptoms, have an impact on their behaviour. Unfavourable temperamental predispositions such as negative emotionality and generally “difficult temperament” contribute substantially to an increased risk of subsequent externalizing and internalizing problems. Our observations corroborate the pronounced main effects of children’s temperament rather than the diathesis stress model of interaction between risky environment and temperamental traits. The decreased predictive power of regulatory problems following the inclusion of temperamental variables points to the mediation effect of temperamental traits in the trajectory between early regulatory difficulties and subsequent behavioural problems.

## Footnotes

1 The project "Nobody slips through the net" (KfdN) is a psychosocial primary and secondary prevention program for families at risk with children in the first year of life. It has been implemented in a total of 11 districts in the German states Hessen, Baden-Württemberg and the whole of Saarland. The key components consist of a course for parents, family home visits mainly through family midwives, and the initiation of a local network with a coordination point for support organisation (detailed in [44]).
